# Supplementary figures and images for: Probing cardiomyocyte mobility with multi-phase cardiac diffusion tensor MRI
Source: PLoS One. 2020 Nov 12;15(11):e0241996. doi: 10.1371/journal.pone.0241996 (PMC7660468; doi:10.1371/journal.pone.0241996)

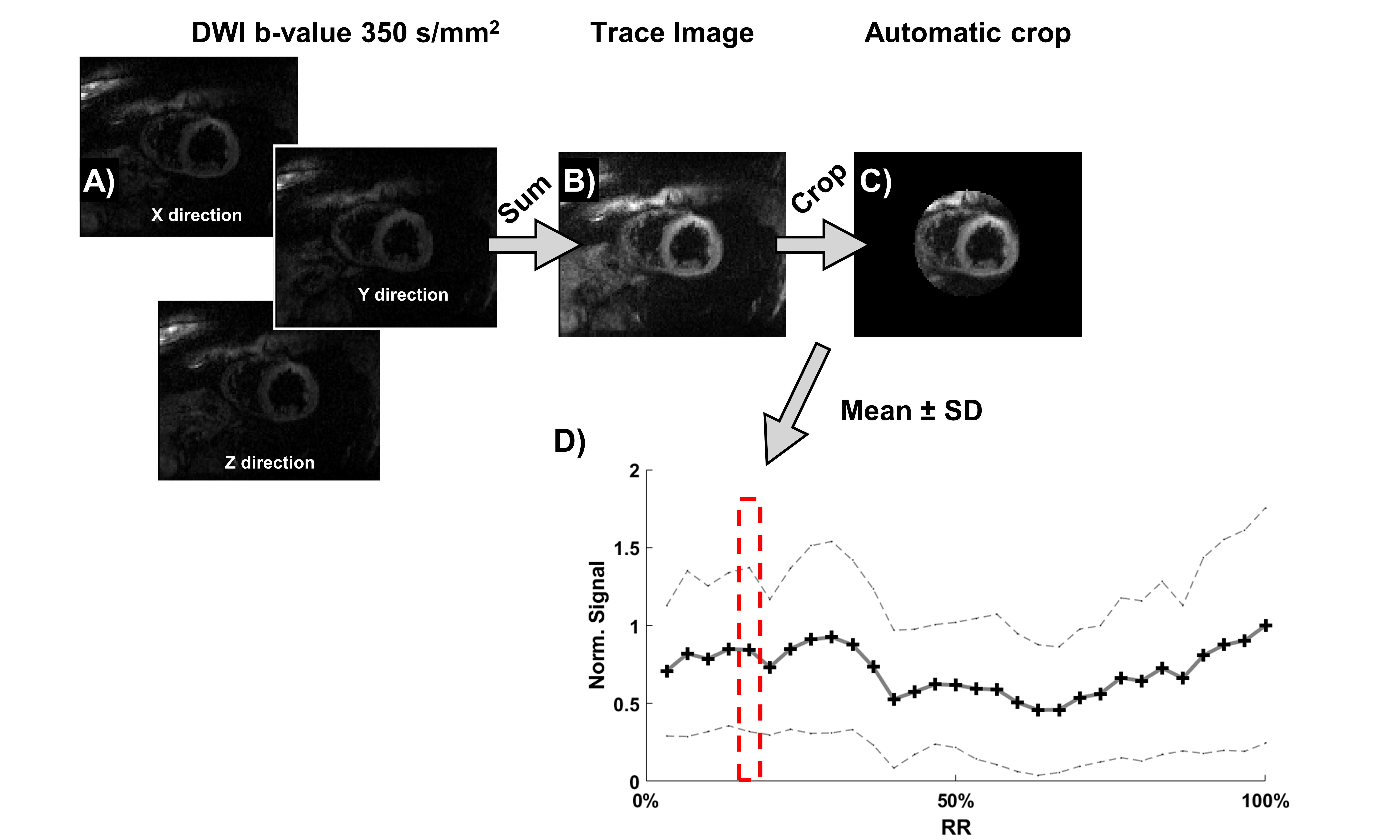

Supplement: S1 Fig — A) Three images corresponding to three diffusion encoding directions x, y, and z are acquired during free breathing for each cardiac phase. B) The three diffusion weighted images are averaged together to generate a trace image. C) The trace image is then circularly cropped with a 48mm radius to only include the central zone of the image corresponding to the heart. D) The mean signal and standard deviation (SD) are computed using the cropped trace image for each cardiac phase. (TIF) [file pone.0241996.s001.tif]

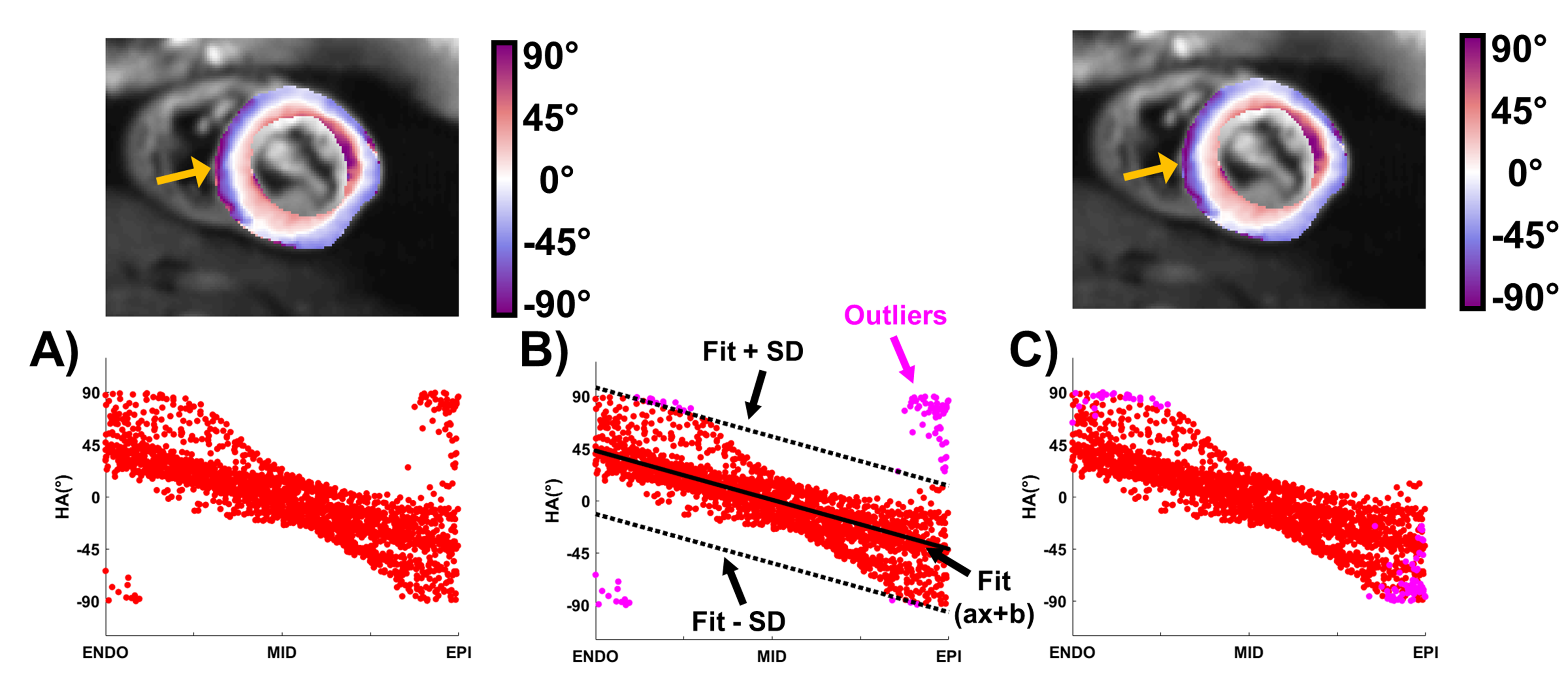

Supplement: S2 Fig — A) The unprocessed distribution of HA as a function of wall depth. B) The HA distribution is fitted to a simple linear model (ax+b) and the data points outside one standard deviation above and below the linear model are classified as outliers. C) Finally, the outliers which are negative from Endo to Mid or positive from Mid to Epi are shifted by 180° in order to flip them in the correct segment. Yellow arrows show the main regions in the cDTI image affected by these outliers. (TIF) [file pone.0241996.s002.tif]

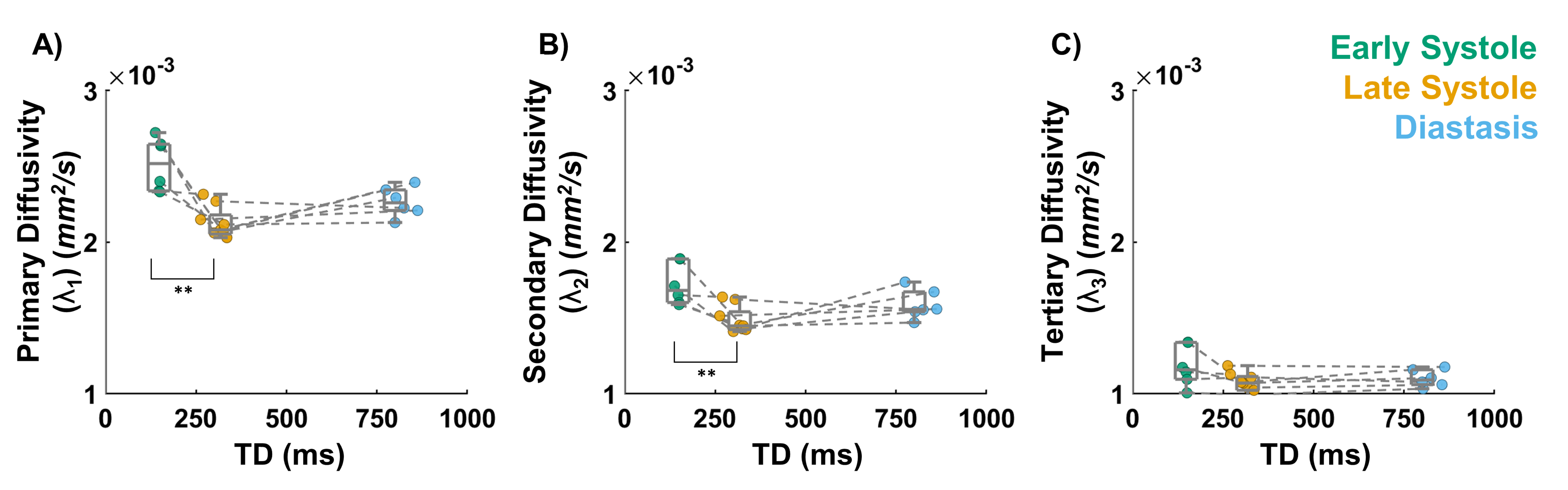

Supplement: S3 Fig — Each volunteer corresponds to a circular marker. Median and IQR are reported across volunteers. (A) Primary diffusivity (λ1), (B) secondary diffusivity (λ2), and (C) tertiary diffusivity (λ3). [** p-value < 0.01] (TIF) [file pone.0241996.s003.tif]

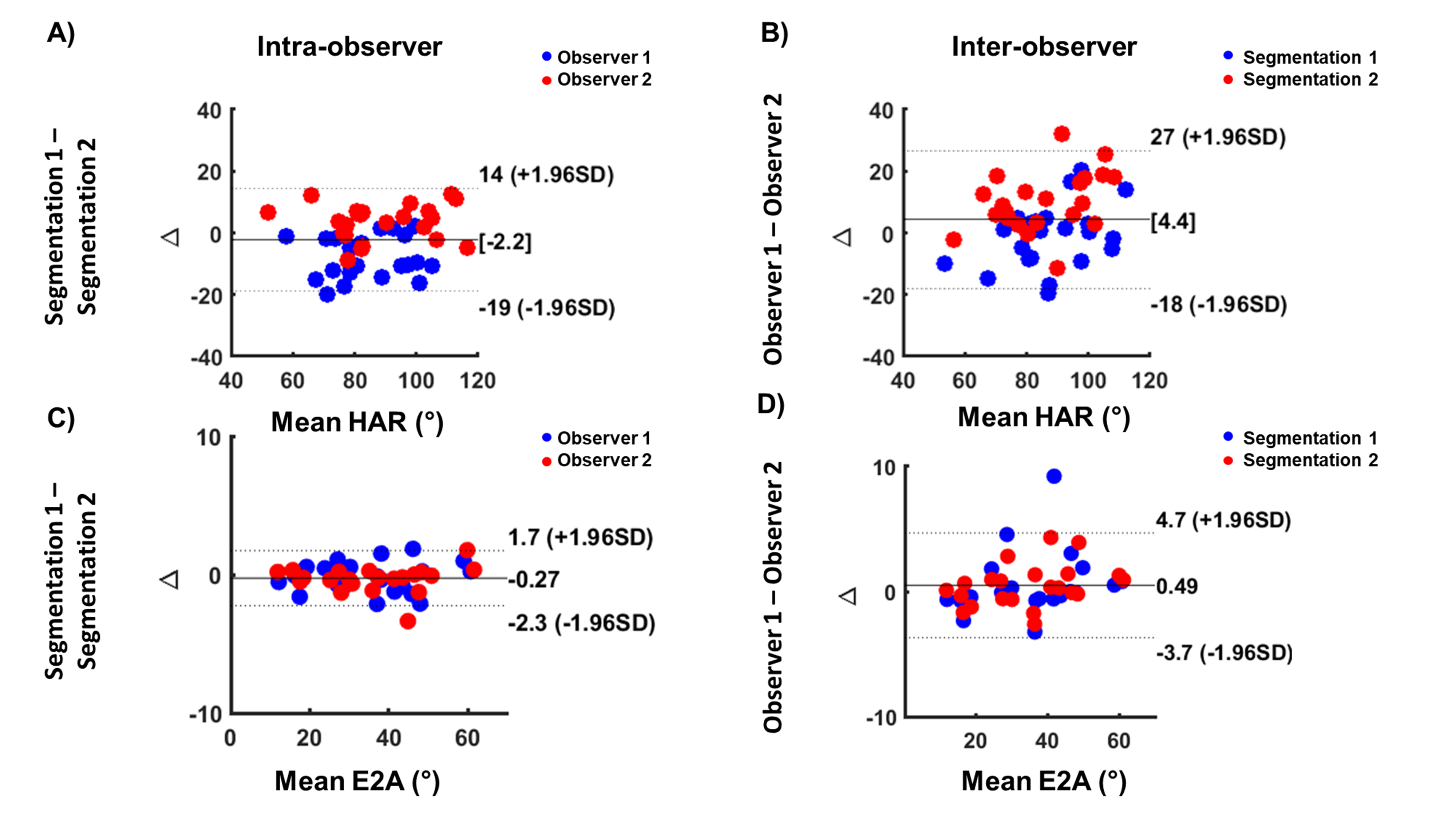

Supplement: S4 Fig — Intra-and inter-observer variability in Helix Angle Range (HAR) (top) and E2 Angle (E2A) (bottom) computed across two segmentations (intra-observer) and two observers (inter-observer). The intraclass correlation coefficient (ICC) for HAR was 76% overall and 84% inter-observer. The intra-observer ICC was 72% for Observer-1 and 92% for Observer-2. The ICC for E2A was 99% overall, inter and intra-observer. (TIF) [file pone.0241996.s004.tif]

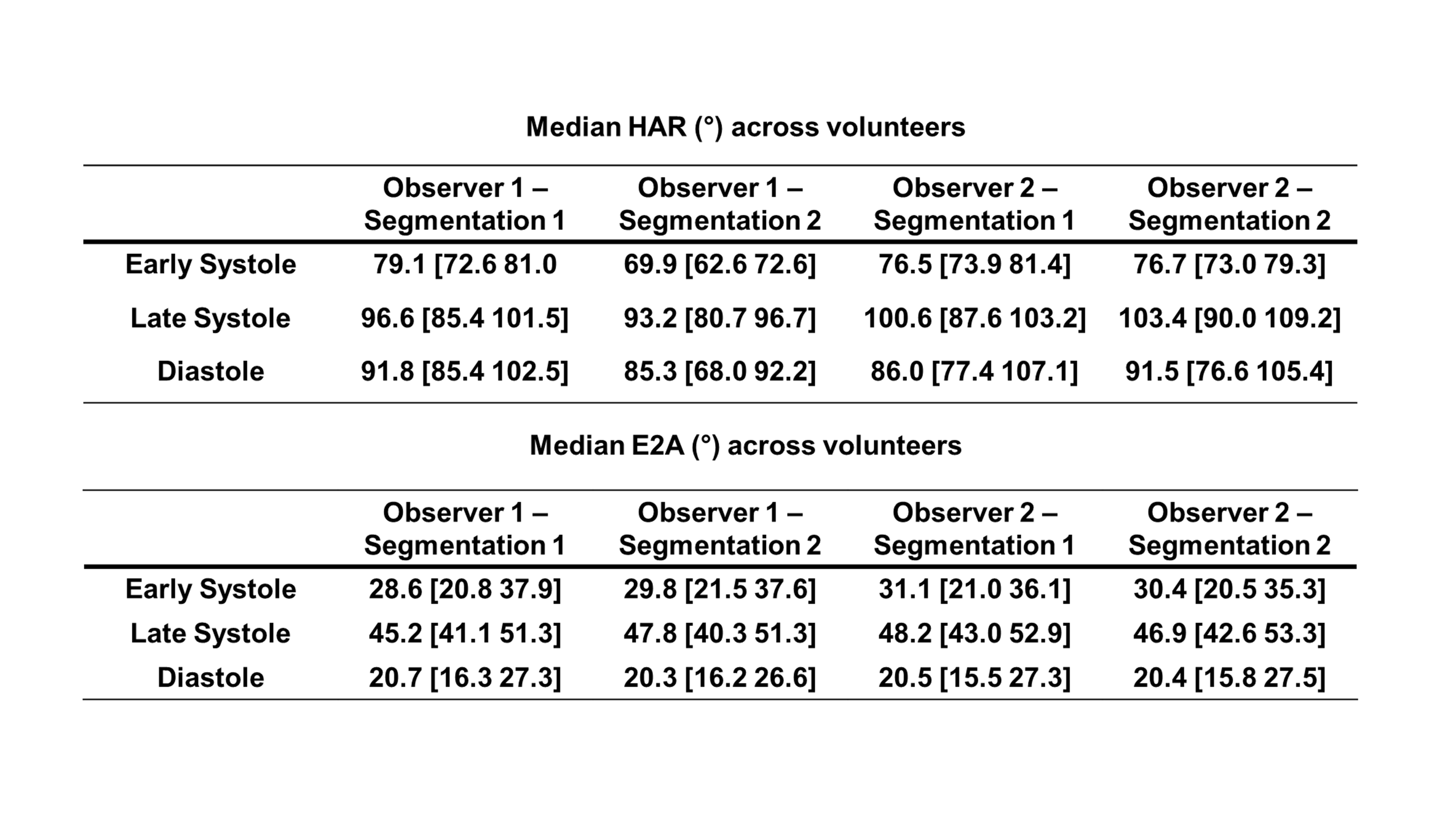

Supplement: S1 Table — Each observer segmented the data twice. Intra-and inter-observer variability does not affect the overall changes in HAR and E2A across the analyzed cardiac phases. (TIF) [file pone.0241996.s005.tif]
