# Supplementary material for: Probing cardiomyocyte mobility with multi-phase cardiac diffusion tensor MRI
Source: PLoS One. 2020 Nov 12;15(11):e0241996. doi: 10.1371/journal.pone.0241996 (PMC7660468; doi:10.1371/journal.pone.0241996)
Supplement: Attachment — Submitted filename: Answer2Reviewers_clean.docx [file pone.0241996.s006.docx]

We appreciate the editor and reviewers’ comments and are grateful that they are experts regarding the subject matter. We have worked to address all concerns with the revisions in our resubmitted manuscript. What follows is a reply to each of the reviewers’ specific comments. **We labeled each comment as “Reviewer 1 Comment 1” (i.e. R1.1)** and these labels are also used to highlight changes in the revised copy of the manuscript. In addition to the response, you will find that surrounding text is provided for context, as needed. Figure added for review are labeled Figure R1, R2. Section, page, and line numbers correspond to the revised PDF proof. References cited in the answers to the reviewers can be found at the end of this document. A clean version of the manuscript has also been provided for convenience. Our replies to each comments are bellow.

Reviewer #1: The Authors present the results of a novel spin-echo sequence to acquire cardiac diffusion tensor imaging (cDTI) with high spatial resolution; three distinct cardiac phases have been chosen using a TD - scout sequence. Overall, the article is interesting and well written.

R1.1) A major limitation (as acknowledged by the Authors) is the through-plane motion between different cardiac phases ("approximately 6.5 mm for a longitudinal strain of -0.15"). Was this longitudinal motion calculated on the long axis cine images? In the discussion, I would suggest the Authors to comment on the possibility of a three-dimensional acquisition instead of a single 2D midventricular short-axis slice, to avoid this limitation.

The longitudinal strain and the corresponding displacement in the Limitation section were given from Moore et al. [1] (reference [37] in the original manuscript).

In the original protocol of this study we did include Strain measurement using two shorts axis 2D DENSE acquisitions with displacement encoding in X, Y and Z. However, DENSE could not be reliably acquired in all our volunteers and was finally not reported in this study. We performed the analysis of the DENSE longitudinal displacement on 5 cases of this study (Figure R1). The Delta displacement between Early/Late systole was -4mm, Late Systole/Diastole was +8mm and Diastole/Early Systole -4mm, which is slightly more than previously stated in the Limitation section. Because the thought-plane displacement is hard to follow using DENSE encoding, please note that these estimations represent likely the worst-case scenario.

We do agree with both reviewers that the through-plane motion remain the major limitation of this work. However the cardiomyocytes distribution is fairly homogenous in the longitudinal direction [2] and thus we do not expect such though plane motion to significantly affect our finding. As mentioned in comment R2.14, the longitudinal motion correction done by Ferreira et al. [3] is very difficult to execute under clinical time. Several other studies investigating cardiomyocyte orientation at multiple cardiac phases did not consider the thought plane motion of the heart [4–6]. We amended the Limitation section to include the element of discussion regarding the longitudinal displacement.

Regarding the single slice acquisition, we agree with the reviewer, a better coverage of the heart would overcome this limitation. Following the reviewer suggestion and comment R2.9, we edited the limitation section to include solutions available to improve the heart coverage.

Limitation, P27-L511-516: “Several approaches have been proposed to improve the spatial coverage without increasing scan time; such as simultaneous multi-slice acquisition [38] or the slice-following navigator technique [24]. Both temporal and spatial coverage could be improved using advanced dual cardiac phase encoding [22]. All these approaches would improve SNR efficiency and could offer improved clinical applicability of the presented protocol.”

Limitation, P27-L517-527: “Second, all three cardiac phases of the cDTI datasets were acquired at the same spatial location for each volunteer. However, the myocardium passes through the imaging plane as the heart contracts and fills and thus the tissue imaged in the three cardiac phases may differ slightly. The mid-ventricular longitudinal motion was approximately -4mm from early to late systole, -4mm from diastasis to early systole and +8mm between late systole and diastasis (estimated using DENSE imaging on a subset of the study subjects). Considering the slice thickness (8mm) and the relative longitudinal homogeneity of the cardiomyocyte orientation [2], it is possible that through-plane tissue motion mildly influences the microstructural mobility reported in this cDTI study. Few multi-cardiac phase cDTI studies in vivo have considered the longitudinal displacement of the heart by estimating this displacement prospectively [3].”

Figure R1: Longitudinal displacement measured across volunteers (N=5) using two shorts axis 2D DENSE Displacement encoding in X, Y and Z direction with a Ke=0.6. Short axis DENSE were immediately above and below the cDTI short axis slices. Solid lines represent the median Z displacement for each of the DENSE acquisition. The dashed line is median longitudinal displacement across volunteers.

R1.2) Moreover, in the discussion I would suggest the Authors to comment on the possibility of acquiring 4 or more TDs, at least under certain conditions (lower heart rate, highly compliant patients...)

We thank the reviewer for this suggestion. It was indeed possible to scan more than 2 cardiac phases during the systolic period for few very compliant volunteers (an example of a 4 cardiac phase (3 Systole + 1 Diastole) acquisition. is given in Figure R2). However, even in these compliant volunteers, the diastasis phase was the only cardiac phase reliable in diastole.

Figure R2: (Top) Example of HA and E2A maps acquired for Early systole (150ms) Mid systole (200ms), Late Systole (270ms) and diastasis (800ms). (Bottom) The corresponding E2A and HAR values reported per cardiac phases.

R1.3) In the introduction (first paragraph), after explaining the HA (E1), the Authors should also briefly define the sheetlet angle (SA, or more precisely referred to as the secondary eigenvector E2 angle); indeed, both parameters are analyzed in the manscript, as depicted in Figure 2. Also the TA (transverse angle) might be cited here.

We want to thank the reviewer for his remark, indeed the introduction lacked in explanation regarding E2A and TA. The Introduction has been modified and now properly introduces E2A and TA.

Introduction, P5-L54-60: “In the left ventricle (LV), the myofiber orientation can be characterized by measuring the helix angle (HA) and the transverse angle (TA). The HA represents the orientation of the myofiber (projected in the local tangent plane) with respect to the circumferential direction. The TA measures the angle between the myofiber direction projected onto the local horizontal plane (normal to the epicardium) and the circumferential direction [2]. . The laminar sheetlet structure of the heart is usually represented by the angle formed between the sheetlet orientation and the cross-myocyte direction [3].“

R1.4) Was there a relationship between image quality and heart rate?

As mentioned in R1.2), even very compliant volunteers, the diastasis phase was the only cardiac phase reliable in diastole as the heart pauses its filling motion. As the heart rate increase, this diastasis phase usually shortens, reducing the window available to scan this cardiac phase. In general, diastole is also more prone to beat-to-beat variation and patient-specific LV motion. This can violate the linear speed/acceleration motion assumption behind the motion compensation technique used in this work. Non-motion compensated waveform combined with an appropriate signal enhancement algorithm such as PCATMP [7, 8] were proven to be efficient in diastole because they have a significantly shorter TE and thus have a much smaller temporal footprint.

In our study specifically, the heart rate pool was 60+/- 11 bpm. We did not find any direct correlation between hearts rates and successfully acquired cardiac phases.

Following this remark and the comment R1.2), we edited the discussion to detail how the heart rate/heart motion affected our image acquisition.

Discussion, P33, L404-416 “The success rates obtained in this work (78%, 100% and 67% in early, late systole and diastasis respectively) are close to the ones found by Scott et al [16] using a similar spin echo M1M2 motion compensation as the one used in the current study. The success rate below 100% in early systole and diastasis shows the limitations of this motion compensation approach. As shown in Fig 3, signal dropout due to cardiac motion was observed, particularly in diastole while most of the systolic phases were available for imaging. The diastolic phase mostly available for imaging corresponded to diastasis, where the LV motion pauses during filing before atrial systole begins. However, usually diastasis shortens as the heart rate increases and beat-to-beat variation in cardiac motion may not respect the linear speed/acceleration assumption behind the M1M2 waveform. Previous works have shown that non-motion compensated waveforms combined with a signal recovery algorithm [25, 26] were able to reliably scan in diastole because of a significantly shorter TE leading to a very small temporal footprint.“

R1.5) Did the Authors perform intra-observer and inter-observer reproducibility of post-processing analysis?

We thank the reviewer for this very important point. We performed an intra/inter observer study to observe the effect of the ROI boundary in the HA/HAR and E2A value we are reporting. Please see the answer R2.4.

R1.6) Did the Authors perform test-retest (inter-study) reproducibility?

Unfortunately at the time of the study we did not planned on acquiring any reproducibility data. Thus we did not re-scan the volunteers scanned for this study.

Reviewer #2: This manuscript describes the use of a motion-compensated spin-echo sequence to acquire relatively high resolution cardiac diffusion tensor imaging (cDTI) data in 9 healthy subjects at multiple cardiac phases. The cDTI data provides measures of cardiac microstructure. Images acquired in early systole, late systole and diastasis were successful in 67%, 100% and 77% of cases respectively. The authors provide helix angle range, sheetlet angle and tensor invariants in multiple cardiac phases. The stated objective of the work was to characterize cardiomyocyte orientations and mobility throughout the cardiac cycle at high spatial resolution and this was achieved.

The study is novel in acquiring in-vivo cDTI data in humans at higher in-plane spatial resolution than previous studies (1.6x1.6mm2), although slice thickness is not reduced relative to comparable studies. The optimal trigger delay for each cardiac phase is determined from a separately acquired novel TD scout. This TD scout allows the user to identify the trigger delays with minimal motion related signal loss from a series of images acquired at a range of TD values. Previous studies by other groups have acquired spin-echo cDTI data at similar cardiac phases without the use of a TD scout, albeit at lower in-plane spatial resolution (Scott et al. JCMR 2018, https://doi.org/10.1186/s12968-017-0425-8). While the TD scout method would appear to be a sensible approach to determining the optimal trigger delay, the success rate at each cardiac phase is not substantially improved over Scott 2018 and the authors do not provide a comparison of the success rate using the TD scout vs. a more standard approach (visual assessment based on cine data). Similarly, the high spatial resolution would seem to be a substantial benefit (if the associated SNR loss is not a problem) when assessing differences in helix angle transmurally and between cardiac phases but no comparison is performed with more typical resolutions to show the benefits.

The authors have included links to a code repository containing the post-processing code, which is commendable. They have also provided a comprehensive list of sequence design parameters, which is excellent.

There are minor typos in the work (included in the comments below), but it is otherwise presented well.

The statistical analysis does not appear entirely appropriate, but should be easily addressed and the PLOS requirements for data sharing do not appear to be satisfied (see specific comments below).

Specific comments:

R2.1) Abstract, results – the success rates for early and late systole appear to be switched around.

We want to thank the reviewer for noticing this important typo. Indeed results have been switched around in the abstract and in the result section. The abstract and result section were modified accordingly.

Abstract, P2-L34-36: “Signal from the patient-specific TD scout sequence demonstrated that SE cDTI acquisition was possible at early systole, late systole, and diastasis in 78%, 100% and 67% of the cases, respectively. “

Result, P13-L235-238: “After visual assessment of the TD scout image quality, it was determined prospectively that cDTI acquisition was possible at late systole, early systole, and diastasis in 100%, 78% (7 out of 9 volunteers), and 67% (6 out of 9 volunteers) of the cases, respectively.”

R2.2) Methods – The text suggests that the EPI readout was 14ms long, while the figure legend (figure 1) says that TEPI was 5ms. It also appears as if TEPI and TEPI-echo are switched around in the legend, but even if so the numbers don’t match the main text.

We thank the reviewer for noticing this incoherence in the main document and in the figure caption. After verification, there was several error in the reported timing. Both text and captions has been edited and now report the correct duration values for EPI.

Figure 1, caption: “The timings of the EPI acquisition T_nav_/δ_90°_/δ_180°_/T_a_/T_b_/T_EPI-echo_/T_EPI_/T_E_ were 8/2/4/24/20/9/23/61ms and the timings of the repeated diffusion encoding gradient δ_1_/δ_2_ were 7.2/12.6ms”

Method, P8-L138-138: “The diffusion encoding duration with motion compensation was 48 ms while the EPI readout was 23 ms”

R2.3) Methods Post-processing – The TD scout images were processed to provide an average image and mean signal. Please clarify what was used to determine the optimal cardiac phases when scanning, was it the quantitative signal intensity or visual appearance?

We agree this was not made clear in the original manuscript. In this work, we used the TD scout in place of a traditional Cine scan to determine the optimal TD available for cDTI. Within predefined range (150-200ms for early systole, 250-350ms for late systole and 700-900ms for diastasis), the TD was chosen solely based on visual appearance (the TD with the fewest artifacts and/or the TD given with the maximum signal intensity). The quantitative analysis of the TD scout acquisition was only retrospective.

We made the Method section more clear regarding this point.

Method, P9-L170-172 “Within these ranges, the TD for each cardiac phase was determined by visual assessment of the Trace signal (average across diffusion directions) of the TD scout acquisition. The TDs were identified by selecting the TD scout images with the least motion induced artifacts.”

Method, P10-L180-183 “For the retrospective quantitative analysis of the TD scout, the mean signal over the central zone of the DWI image was reported (see S1 Fig). Subsequently, the reported mean signals were normalized with respect to the maximum mean signal across all cardiac phases per volunteer.”

R2.4) Methods Quantitative analysis – There is much focus in the manuscript on helix angle range (HAR). However, I expect that HAR is highly dependent on the definition of the epi and endocardial borders drawn and therefore could be rather observer dependent. I suggest that additional analysis to provide intra and inter observer variability of the HAR measures used should be performed.

Following comment R1.5), we fully agree with both reviewers, the angular values reported in this manuscript namely HA, HAR, E2A and TA are dependent of the manually drawn Epicardial border of the LV. The HA, in particular, varies transmurally in the LV which makes its quantitative analysis challenging and relatively observer dependent. On the other hand E2A/TA are reported across the entire LV and thus intra/inter-observer variation are strongly mitigated.

Two metrics can be found in literature regarding the quantitative analysis of HA: HAR and HA slope. The latter has the advantage of being more independent of the boundary definition of the LV, however this metric only makes sense for linear transmural distributions of the HA. In this work, we opted for the HAR metric as it does not make any assumption of the HA distribution as we observed different distribution patterns. However, HAR is more observer dependent because it considers only the cardiomyocyte orientation close to the Endocardium and Epicardium border which are manually determined.

We performed an inter and intra observer study where we compared two observers each performed each two measures of the HAR and E2A. In Figure R3, we can see that the same trend for HAR and E2A was measured for both observer and both measures. In Figure R4, we observe the intra and inter observer variations for E2A and HAR. For intra-observed and inter-observed, HAR presented a Standard deviation around 10° and a bias around 5° degree across volunteers. The intraclass correlation coefficient (ICC) for HAR was 76% overall and 84% for the inter-observer analysis. The intra-observer ICC was 72% for observer 1 and 92% for observer 2. For E2A no bias was observed and the SD was below 5° while the ICC was close to 99% overall and inter-intra-observer. Despite these observer variations per subject, the median across volunteer remain relatively unchanged as shown in table TR1.

We included the Bland-Altman and the result table to the supplementary material of the manuscript. We have also included a paragraph in the discussion to comment on the observer dependence of the HAR result we are reporting here.

Discussion, P25-L468-475: “Finally, the angular metric used to represent the microstructure (namely HA, TA, E2A, HAR) are based on circumferential and radial directions constructed from manually defined segmentations. This inter-observer dependence is particularly significant in evaluation the HAR metric based on the HA in proximity of the endocardial and epicardial surfaces. This can be seen in the intra/inter observer study presented in S4 Fig, where we report more variation across subjects for HAR than E2A. However as shown in S1 Table, median trends during the cardiac cycle remain consistent across intra and inter trials.“

Figure R3: Angle statistics at early systole, late systole, and diastasis across all volunteers obtained for each observers and each of their segmentations. Each volunteer corresponds to a circular marker. Median and IQR are reported across volunteers. (Top) Helix Angle Range (HAR), (Bottom) E2 Angle (E2A).

Supplementary Figure 4: Intra-and inter-observer variability in Helix Angle Range (HAR) (top) and E_2_ angle (E2A) (bottom) computed across two segmentations (intra-observer) and two observers (inter-observer). The intraclass correlation coefficient (ICC) for HAR was 76% overall and 84% inter-observer. The intra-observer ICC was 72/% for observer 1 and 92% for observer 2. The ICC for E2A was 99% overall, inter and intra-observer.

Table TR1: Median Helix Angle Range (HAR) and E_2_ angle (E2A) computed by two observers across volunteers. Each observer segmented the data twice. Intra-and inter-observer variability does not affect the overall changes in HAR and E2A across the analyzed cardiac phases.

R2.5) Methods – statistical analysis: The data are presented as medians and interquartile ranges, but the statistical comparisons are parametric. The use of median and interquartile range, implies non-parametric data. On what basis were parametric tests used? If the use of parametric tests can be justified then data should be presented as mean and standard deviation. Furthermore, it is usual to perform an ANOVA type test to compare more than two groups and then perform subsequent paired comparisons where differences are identified on ANOVA.

We want to thank the reviewer for noticing this incoherence. We originally used parametric test because of their statistical power compared to the non-parametric ones but the reviewer is correct, this is incorrect since we are using a non-parametric representation of our data. We did opt for this non-parametric representation for multiple reason: First our samples size is low (maximum <9); second our samples size vary across cardiac phases (N=7,9,6) ; and finally most of our metric represent angle with an unknown distribution type.

We have re-processed the statistical analysis using SPSS (version 29, IMB Corp.) with a non-parametric one-way ANOVA - Kruskal-Wallis H-test across cardiac phases. The corresponding pairwise comparisons were performed when the overall ANOVA hypothesis was rejected.

We accordingly edited the result/discussion section as well as figure 7, 8 and supplementary figure 3. The method now reads:

Method, P12-L222-226: “All statistical analysis were carried out in SPSS (Version 26, IBM Corp.) using a non-parametric Kruskal-Wallis one-way ANOVA. For statistically different distributions, corresponding paired wise comparisons were realized between cardiac phases using a Bonferroni post-hoc correction. p<0.05 was considered statistically significant.”

R2.6) Methods – last sentence, typo “Holm-Bonferroni and correction”.

We thank the reviewer for noticing this typo. Edited.

R2.7) Results – It’s unclear where statistical comparisons have been performed and differences found to be non-significant. I suggest quoting p-values for all comparisons performed, whether the result is significant or not.

Following comment R2.5), we re-processed the entire statistical analysis and reworked the result section. Now, at the end of each major paragraph in the result section, we have included all the statistical comparison between all parameters as well as all corresponding p-value. We believe this change has improved the overall clarity and presentation of the results.

Results, P18-L319-322: “Overall, statistical differences were found in HAR across cardiac phase (p=0.004). Paired wised comparison were significant between early to late systole (p=0.005) and between diastasis and early systole (p=0.004), but no statistical differences were detected between late systole and diastasis (p=1). ”

Results, P18-L332-336: “Overall, TA distributions were statistically different among cardiac phases (p=0.033), but no paired-wise difference were observed after post-hoc correction between early and late systole (p=0.094), late systole and diastasis (p=1) and diastasis and early systole (p=0.054). ”

Results, P19-L339-342: “Across cardiac phases, E2A distributions were statistically different (p=0.001). After post-hoc correction paired wise statistical differences were only found between late systole and diastasis (p=0.001), but not between early and late systole (p=0.065) nor diastasis and early systole (p=0.576). ”

Results, P19-L554-559: “Overall, statistical differences were found across cardiac phase for MD (p=0.006), λ1 (p=0.002), λ2 (p=0.009) but not for λ3 (p=0.153), λ2/1 (p=0.54) or FA (p=0.32). For MD, λ1 and λ2, respectively paired-wised comparisons showed significant statistical differences between early and late systole (p=0.005, p=0.002, p=0.009) but not for late systole and diastole (p=0.293, p=0.166, p=0.193) nor diastole and early systole (p=0.589, p=0.562, p=1).”

R2.8) Results – Differences in helix angle and helix angle range are presented for 6 AHA segments. Some of these differences could be the consequence of off-resonance effects rather than changes in microstructure. These off-resonance effects are generally most severe in the inferolateral wall of the LV and least severe in the septum.

We want to thank the reviewer for this suggestion. Indeed the inferolateral wall of the LV was more subject to off-resonance and associated distortion. However, we are not sure on what measure the off-resonance participated to the HAR/E2A changes observed in this particular cardiac segment. It was recently demonstrated by Van Gorkum et al. [9] that in case of compression there is a slight decrease in MD and more pronounced decrease of FA. Even with very pronounced distortion, as shown in the figure R5, we did not observed local change in MD nor FA.

We included a comment in the Discussion section regarding distortion in the inferolateral segment.

Figure R5: Example of the diffusion weighted Trace image for one volunteer and the corresponding Mean Diffusivity (MD) and Fraction of Anisotropy (FA) maps. The red arrow indicates a severe off-resonance distortion in the Trace image, but no significant difference are observed in the MD and FA in the distorted region compared to the remote region.

Discussion, P23-L422-426: “In this study, high spatial resolution enabled the detailed analysis of in vivo HA per AHA segment, revealing distinct microstructural organization that varies per-segment. However high resolution remains challenging, particularly in the inferolateral segment which is prone to strong geometrical distortion due to the lung/heart/liver interface [28].”

R2.9) Discussion – The spin-echo sequence is theoretically much more SNR efficient than the STEAM sequence, but the calculations are typically based on a TR of 1 or 2 cardiac cycles. This study uses a TR=4 RR intervals which is very long and results in a lot of dead time. Can this be improved in future studies by interleaving slices? Presumably in most end expiratory periods there are two cardiac cycles, which could be used to acquire 2 slices with no loss of SNR if angled refocussing pulses are used (as in Stoeck 2014 <https://doi.org/10.1371/journal.pone.0107159>)

This study was took a conservative approach as a single slice was acquired with a long repetition time (TR=4RR). This long TR likely benefited our acquisition strongly in terms of SNR, which in part allowed the high resolution acquisition. However, the reviewer is correct, this protocol was not highly SNR efficient in comparison to literature. We did mention the limited slice coverage of our study in the limitations section but we did not express it in term of SNR efficiency. The multi-slice approach proposed by Stoeck et al. [5] to acquire two cardiac phases simultaneously is indeed highly time efficient and should be considered. We edited the limitations section to include element about the SNR efficiency and solutions available to improve them, including the approach of Stoeck et al.

Limitation, P27-L509-516: “A first limitation of this study is the single-slice heart coverage acquired with a long TR to maximize SNR. However this limits characterizing cardiomyocyte mobility throughout the heart and provides a poor overall SNR efficiency. Several approaches have been proposed to improve the spatial coverage without increasing scan time; such as simultaneous multi-slice acquisition [38] or the slice-following navigator technique [24]. Both temporal and spatial coverage could be improved using advanced dual cardiac phase encoding [22]. All these approaches would improve SNR efficiency and could offer improved clinical applicability of the presented protocol.”

R2.10) Discussion – median missing from E2A values quoted in late systole.

We thank the reviewer for noticing this typo. The median value was added accordingly.

R2.11) Discussion – The variation in E2A and HA between studies is attributed to spatial resolution, which is rather speculative and doesn’t account for other potential contributing factors such as the diffusion time, sensitivity to off-resonance etc.

We agree with the reviewer, the variation of E2A/HA observed between studies is very likely a combination of various factors. For instance, most of the work *in vivo* from literature cited in this paragraph report E2A and HA using a STEAM approach where a spin-echo approach was used here. These approaches differ indeed in term of off-resonance, SNR but mostly in term of b-values and diffusion time, which should strongly affect the sensitivity, and accuracy of second and third EigenVector E2/E3. These factors contribute to the differences observed between studies. However, we still believe that spatial resolution is one of the major factors contributing to these differences. As shown by McClymont et al [10], specifically studying HAR, typical clinical resolution can underestimate HAR by 18° in mid-ventricle and up to 30° in base and apex.

The reviewer is correct, the design of our study doesn’t allow us to claim that high resolution provides a better depiction of the microstructure and thus the paragraph in the Discussion section was indeed too speculative. We edited it to include the element of discussion mentioned above and comments from R2.4.

Discussion, P23-L417-426: “Typical in-plane spatial resolutions in clinical cDTI studies range from 2x2 mm2 to 3x3 mm2, usually limited by low SNR. Because HA varies transmurally, higher spatial resolution could play an important role in improving the assessment of regional cardiomyocyte orientation and mobility. Previously McClymont et al. [27] have demonstrated that typical spatial resolutions could underestimated the HAR by up to 18° in part due to partial volume effects. In this study, high spatial resolution enabled the detailed analysis of in vivo HA per AHA segment, revealing distinct microstructural organization that varies per-segment. However high resolution remains challenging, particularly in the inferolateral segment which is prone to strong geometrical distortion due to the lung/heart/liver interface [28].”

Discussion, P25-L463-468: “Although HAR and E2A mobility trends are consistent across cDTI studies, there is a broad range in the reported magnitude of HAR and E2A changes during contraction and filling. As shown by Scott et al [16], these variations could be attributed in part to difference in sensitivity (diffusion time) between SE and STEAM imaging. In addition, a collection of artifacts (motion, off-resonance, chemical shift of fat, etc.) are also present in cDTI, with a different effect in SE or STEAM imaging [9, 16, 22, 35].”

R2.12) Discussion – typo “were observe[d] among different cardiac phases”.

Thank you for noticing this typo. Edited.

R2.13) Discussion – The discussion of the reasons behind the changes in tensor invariants between cardiac phases is slightly confusing. If the M1-M2 compensated gradient waveforms are insensitive to blood flow then wouldn’t the signal in both the reference (b=0) and diffusion encoded images (b=350) be equally affected by blood flow? This would make the diffusion tensor invariant to changes in blood flow.It’s also not clear what the authors think causes the changes in parameters if it is not caused by changes in the microscopic compartments. Presumably the sequence is somewhat sensitive to changes in compartment dimensions otherwise the signal would be isotropic?

We agree with the reviewer, the original paragraph was very unclear. We have not yet a clear explanation of why we found this higher diffusivity (MD, λ1, λ2) in early systole compared to late systole or diastasis. By looking more deeply in the literature, we could not find any clear trend neither. In the work of Stoeck et al. [5], a higher MD was found in systole 1.2±0.2 x 10^-3^mm^2^/s than diastole 0.9±0.1 x 10^-3^mm^2^/s using a STEAM approach. Scott et al. [4] found the opposite trend with an MD of 1.02 [0.14] x 10^-3^mm^2^/s in systole, 1.07 [0.12] x 10^-3^mm^2^/s at the systolic sweet spot (roughly mid systole) and 1.13 [0.08] x 10^-3^mm^2^/s in diastole using a STEAM approach. In the same study using a SE sequence they found an MD of 1.46 [0.43] x 10^-3^mm^2^/s in systole, 1.70 [0.18] x 10^-3^mm^2^/s at the systolic sweet spot and 1.78 [0.34] x 10^-3^mm^2^/s in diastole. In our previous work using SE [11], we did find a higher MD in diastole 1.91 ± 0.34 × 10^−3^mm^2^/s compared to systole 1.58 ± 0.09 × 10^−3^ mm^2^/s. In another work of Stoeck et al. [12] using a SE with M1M2 waveform, cardiac phases in very early systole presented more motion artifact leading to an artificial increase of MD.

We have included bellow in figure R6, the eight repetition RAW data for b-value=0 and b-value=350 s/mm^2^ at one direction. This example displays all the types of motion artifacts we could find in our acquisition. The blue and red arrow show residual cardiac bulk motion that is sometimes seen in the diffusion weighted images. These artifacts are usually spatially located, however as shown in Figure R6 the MD maps are globally increased. Another artifact highlighted by the green arrows can be seen in the non-diffusion weighted image. This type of artifact is likely due to physiological motion or false ECG triggering which lead to an early acquisition, reducing the TR and due to T1 effect leading to a lower signal pool available for imaging. This lower signal would indeed generate artificially lower MD maps overall. However, this type of artifact is not very frequent in our dataset and cannot by itself explain the trend we are seeing.

Regarding the blow flow effect, motion compensated waveforms refocus some of the blood in the cavity even with a moderate b-value of 350s/mm^2^. As shown in Figure R6, this blood pool differs from non-weighted compared to diffusion weighted and from Early systole to Late systole. However, this alone would lead to an artificial higher MD only in the endocardium portion of the heart and we observed an overall increase of MD across the LV.

Still regarding blood flow, we mentioned in the discussion that capillaries are filled with blood during Early systole which could affect the diffusion signal through the IVIM effect. IVIM can be decomposed in two family of effects: 1) incoherent flow in small vessels changing direction frequently and 2) coherent flow in randomly distributed vessels. We demonstrated in our previous work [13] that motion compensated waveforms compensate the latest scenario only and not the first one. However, this work was done in Liver which contains a different micro-perfusion architecture compared to the heart. We believe its still too early to draw conclusions on the heart from our previous work.

Finally, regarding the microstructural sensitivity, we intended to clarify that the diffusion time of the SE approach was too small to detect variation of the microstructure between two cardiac phases. For instance, the change in cardiomyocyte diameter between contraction and rest. With a long mixing time, the STEAM approach is probably more sensitive to this type of changes.

We rewrote this paragraph to include the element discussed here.

Discussion, P26-l486-507: “The MD and FA values reported in this work are in the range of previously reported values in healthy volunteers using spin-echo sequences. Statistically higher MD values were found at early systole 1.79 [1.69, 1.92] x 10-3 mm2/s compared to late systole 1.53 [1.5, 1.62] x 10-3 mm2/s or diastasis 1.64 [1.62, 1.69] x 10-3 mm2/s. In a previous work by Stoeck et al. [22], a higher MD was found in systole 1.2 ± 0.2 x 10-3 mm2/s compared to diastole 0.9 ± 0.1 x 10-3 mm2/s using a STEAM approach. Scott et al. [16] .found the opposite trend with an MD of 1.02 [0.14] x 10-3 mm2/s in systole, 1.07 [0.12] x 10-3 mm2/s at the systolic sweet spot and 1.13 [0.08] x 10-3 mm2/s in diastole using a STEAM approach. In the same study using a SE sequence, MD was 1.46 [0.43] x 10-3 mm2/s in systole, 1.70 [0.18] x 10-3 mm2/s at the systolic sweet spot and 1.78 [0.34] x 10-3 mm2/s in diastole. In our previous work [36] using SE, a higher MD was found in diastole 1.91 ± 0.34 x 10-3 mm2/s compared to systole 1.58 ± 0.09 x 10-3 mm2/s. Values were reported as mean ± standard deviation, median [Intra Quartile Range] or median [Quartile 1, Quartile 3]. The source of this increase could be explained by several effect. The higher MD in early systole could be partially due to micro-perfusion, which is known to increase during filling and whose signal could not be fully spoiled by the motion compensated diffusion encoding gradients [37]. Despite our use of a motion compensated waveform, unwanted cardiac bulk motion present in early systole could artificially increase MD [15]. However, in this work, the diffusivity differences are unlikely due to a change in cellular/extra-cellular compartment between cardiac phases. The b-value (350 s/mm2) and the diffusion encoding time used here (Δ =40ms) were too small to sensitively capture these compartmental changes [16], as it is also confirmed by fact that the ratio λ2/1 remains constant across cardiac phases.”

Figure R6: Example of RAW images for one volunteer at early systole and late systole. The eights repetitions for the non-diffusion-weighted (b0) and the first direction (Dir. 1) of the diffusion weighted (b350) are represented.

R2.14) Discussion, final paragraph – Several studies including Ferreira et al. JCMR 2014 https://doi.org/10.1186/s12968-014-0087-8 have shifted the imaging plane between cardiac phases to account for this potential issue.

The reviewer is correct; we did miss this information in the study of Ferreira et al. [3]. We amended the Limitation section according this comment and the element given in R1.1).

Limitation, P27-L517-527: “Second, all three cardiac phases of the cDTI datasets were acquired at the same spatial location for each volunteer. However, the myocardium passes through the imaging plane as the heart contracts and fills and thus the tissue imaged in the three cardiac phases may differ slightly. The mid-ventricular longitudinal motion was approximately -4mm from early to late systole, -4mm from diastasis to early systole and +8mm between late systole and diastasis (estimated using DENSE imaging on a subset of the study subjects). Considering the slice thickness (8mm) and the relative longitudinal homogeneity of the cardiomyocyte orientation [2], it is possible that through-plane tissue motion mildly influences the microstructural mobility reported in this cDTI study. Few multi-cardiac phase cDTI studies in vivo have considered the longitudinal displacement of the heart by estimating this displacement prospectively [3].”

R2.15) Figure 3 – this seems to be identical to supplementary figure 3 apart from the normalisation of the x axis in the supplementary figure. The normalised axis seems more logical in my opinion and the inclusion of both seems excessive.

We fully agree with the reviewer, we accordingly removed the additional figure 4 and replaced figure 3 with the normalized one.

R2.16) Data availability – PLOS one requires that a minimal data set is shared (https://journals.plos.org/plosone/s/data-availability). It does not appear to me that this is the case for this manuscript.

“For example, authors should submit the following data:

- The values behind the means, standard deviations and other measures reported;

- The values used to build graphs;

- The points extracted from images for analysis.”

We added a link to download the database of this study however due to a privacy conflict with our institution we could not directly provide the dicom images.

In this link can be found in a matlab format: all RAW data, the data saved at each intermediate state of the reconstruction process and finally the fully reconstructed and process dataset (including all 4 ROIs and the corresponding angular maps). A matlab script is also provided that was used to analyze the dataset and generate the figures of this manuscript. The link also contains in an excel format the individual median extracted per volunteer and the overall reported median and corresponding statistic.

Method, P10-L177-179: ”All analyses and cDTI reconstruction were performed off-line using custom software (Matlab, Mathworks, repository: https://github.com/KMoulin/DiffusionRecon). Raw and reconstructed dataset can be found at https://med.stanford.edu/cmrgroup/data.html.”

**References**

1.	Moore CC, Lugo-Olivieri CH, McVeigh ER, Zerhouni EA (2000) Three-dimensional Systolic Strain Patterns in the Normal Human Left Ventricle: Characterization with Tagged MR Imaging. Radiology 214:453–466

2.	Lombaert H, Peyrat J, Croisille P, Rapacchi S, Fanton L, Cheriet F, Clarysse P, Magnin I, Delingette H, Ayache N (2012) Human Atlas of the Cardiac Fiber Architecture: Study on a Healthy Population. IEEE Transactions on Medical Imaging 31:1436–1447

3.	Ferreira PF, Kilner PJ, McGill L-A, et al (2014) In vivo cardiovascular magnetic resonance diffusion tensor imaging shows evidence of abnormal myocardial laminar orientations and mobility in hypertrophic cardiomyopathy. Journal of Cardiovascular Magnetic Resonance. https://doi.org/10.1186/s12968-014-0087-8

4.	Scott AD, Nielles-Vallespin S, Ferreira PF, Khalique Z, Gatehouse PD, Kilner P, Pennell DJ, Firmin DN (2018) An in-vivo comparison of stimulated-echo and motion compensated spin-echo sequences for 3 T diffusion tensor cardiovascular magnetic resonance at multiple cardiac phases. J Cardiovasc Magn Reson 20:1

5.	Stoeck CT, Kalinowska A, von Deuster C, et al (2014) Dual-Phase Cardiac Diffusion Tensor Imaging with Strain Correction. PLoS ONE 9:e107159

6.	Nielles-Vallespin S, Khalique Z, Ferreira PF, et al (2017) Assessment of Myocardial Microstructural Dynamics by In Vivo Diffusion Tensor Cardiac Magnetic Resonance. Journal of the American College of Cardiology 69:661–676

7.	Rapacchi S, Wen H, Viallon M, Grenier D, Kellman P, Croisille P, Pai VM (2011) Low b-Value Diffusion-Weighted Cardiac Magnetic Resonance Imaging: Initial Results in Humans Using an Optimal Time-Window Imaging Approach. Investigative Radiology 46:751–758

8.	Wei H, Viallon M, Delattre BMA, Moulin K, Yang F, Croisille P, Zhu Y (2015) Free-Breathing Diffusion Tensor Imaging and Tractography of the Human Heart in Healthy Volunteers Using Wavelet-Based Image Fusion. IEEE Transactions on Medical Imaging 34:306–316

9.	Gorkum RJH van, Deuster C von, Guenthner C, Stoeck CT, Kozerke S Analysis and correction of off-resonance artifacts in echo-planar cardiac diffusion tensor imaging. Magnetic Resonance in Medicine. https://doi.org/10.1002/mrm.28318

10.	McClymont D, Teh I, Schneider JE (2017) The impact of signal-to-noise ratio, diffusion-weighted directions and image resolution in cardiac diffusion tensor imaging – insights from the ex-vivo rat heart. Journal of Cardiovascular Magnetic Resonance. https://doi.org/10.1186/s12968-017-0395-x

11.	Aliotta E, Moulin K, Magrath P, Ennis DB (2018) Quantifying precision in cardiac diffusion tensor imaging with second-order motion-compensated convex optimized diffusion encoding. Magn Reson Med 80:1074–1087

12.	Stoeck CT, Deuster C von, Genet M, Atkinson D, Kozerke S (2016) Second-order motion-compensated spin echo diffusion tensor imaging of the human heart. Magnetic Resonance in Medicine 75:1669–1676

13.	Moulin K, Aliotta E, Ennis DB (2019) Effect of flow-encoding strength on intravoxel incoherent motion in the liver. Magnetic Resonance in Medicine 81:1521–1533
