# Supplementary material for: Probing cardiomyocyte mobility with multi-phase cardiac diffusion tensor MRI
Source: PLoS One. 2020 Nov 12;15(11):e0241996. doi: 10.1371/journal.pone.0241996 (PMC7660468; doi:10.1371/journal.pone.0241996)
Supplement: Attachment — Submitted filename: Answer2Reviewers_clean.docx [file pone.0241996.s007.docx]

We appreciate the editor and reviewers’ second round of comments and we have addressed all new concerns with the revisions in our resubmitted manuscript. What follows is a reply to each of the reviewers’ specific comments. **We labeled each comment as “Reviewer 1 Comment 1” (i.e. R1.1)** and these labels are also used to highlight changes in the revised copy of the manuscript. In addition to the response, surrounding text is provided for context, as needed. Figures added for the review are labeled as Figure R1, R2, etc. Section, page, and line numbers correspond to the revised PDF proof. References cited in the answers to the reviewers can be found at the end of this document. A clean version of the manuscript has also been provided for convenience. Our replies to each comments are bellow.

**Reviewer #2: Thank you for revising the manuscript based on my comments and those of the other reviewer. I appreciate the time and effort that you have gone to in addressing my concerns. I feel that the added intra and inter-observer analysis of HAR and E2A is a really good addition to the manuscript. I also appreciate the provision of the associated data and analysis code. I have a few additional comments mostly related to the additional results added to the paper that I hope can be addressed.**

**R2.1) The intra and inter observer reproducibility study is interesting, but should be described in the methods and results rather than being first mentioned in the discussion. The same principle applies to the DENSE study and analysis, which should be described in the methods and results.**

We thank the reviewers for suggesting the addition of the intra/inter-observer analysis. As the reviewer suggested we have added both the short DENSE study and the reproducibility study to the Methods and Results sections.

Methods, P25-L468-475: “A short-axis cine DENSE sequence (resolution 2.3 x 2.3 mm, 3D four-point displacement encoding, k_e_=0.8, TE=1.05ms, TR=15ms, 30 cardiac phases or more) was also performed using free-breathing and an expiratory triggered navigator for a subset of subjects (N=5). The measured displacements were used to estimate the longitudinal cardiac displacement between the cardiac phases of interest.”

Methods, P25-L468-475: “An intra- and inter-observer study was performed to estimate the reproducibility of HAR and E2A. Two manual segmentations (intra-observer) were performed by two observers (inter-observer) for a total of four observations per subject. Median HAR, E2A as well as intra-class correlation coefficient (ICC) were reported across subjects for the four observations. The first segmentation from the first observer was used for all non-reproducibility analyses reported in the results section.”

Results, P25-L468-475: “Due to longitudinal cardiac motion, the three imaged cardiac phases may have been acquired at slightly different positions along the LV long axis. Using the DENSE acquisition from a subset of the volunteers (N=5), the mid-ventricular longitudinal motion was estimated to be 4 mm from early to late systole, 4 mm from diastasis to early systole, and -8 mm between late systole and diastasis (positive values correspond to motion from base towards apex).“

Results, P25-L468-475: “Intra- and inter-observer analyses were performed using two observers, each segmenting two sets of LV ROIs. Intra- and inter-observer variations for E2A and HAR are displayed in Fig. S4. The ICC for HAR was 76 [61, 88] % overall and 84 [73, 91] % for the inter-observer analysis. The intra-observer ICC was 72 [45, 87] % for Observer 1 and 92 [83, 97] % for Observer 2. For E2A values, the ICC was 99% overall, across intra- and inter-observer analyses. Median values for E2A and HAR for the three cardiac phases per segmentation are given in Supplementary Table 1. Intra- and inter-observer variations did not affect the overall median nor the trends of E2A and HAR across cardiac phases.”

**R2.2) Based on figure 3 and line 239, it appears that the early systolic time point was the same trigger delay index (nth TD) for all acquisitions. In which case the TD scout serves more as a method to determine whether cDTI will be possible in a particular phase than to select the correct phase? In that case, the TD scout serves to save time when it demonstrates that cDTI data cannot be acquired at a particular trigger delay, which could be highlighted in the discussion.**

The reviewer is correct, the early systolic images were acquired at roughly the same time point 150 [148, 153] ms across volunteers. Please note that Figure 3 uploaded during the previous review round was mistakenly not normalized with respect to each subjects’ RR interval. We have now added a normalized version of Figure 3.

Regarding the use of the TD scout, there is a difference between what we did for this study and what is typically done in clinical cDTI studies. Fundamentally, the TD scout is a quick cine scan of diffusion. In this work, we studied 3 cardiac phases with imposed boundaries (Early systole, TD~150-200 ms; Late systole TD~250-350 ms; Diastasis TD~700-900 ms) and we selected the best TD within these boundaries. The boundaries were simply imposed in order to have cDTI at three nearly similar cardiac phases across volunteers. In a clinical setting, radiologists only acquire a single cardiac phase of cDTI and thus the TD scout is used in a slightly different manner. MR technicians run the TD scout prospectively only during the systolic phase (0~500ms) and select the systolic cardiac phase that provides the most signal without motion artifact. They usually pick the cardiac phase which is the closest to peak contraction because the cardiac wall is the thickest. Overall this results in a net gain of time for the protocol workflow because the cDTI is always performed at the best possible cardiac phase. There is no long and continuous iterations to select the appropriate cardiac phase.

As a note, the diastolic period is of particular clinical interest, as quantitative CMR imaging is typically realized in diastole (T1/T2/ECV mapping). However, diastole is even less reliable for cDTI using M_1_M_2_ waveforms in patients because they tend to have a faster heart rate and a short or absent diastasis phase. We have amended the discussion with an element of the response above:

Discussion, P25-L468-475: “The TD scout calibration was designed to efficiently identify cardiac phases with little signal dropout for each subject. This yields a fast tool for prospectively determining which cardiac phases are best suited for diffusion weighted imaging. As a prospective tool, the TD scout reduces exam time as it ensures that the subsequent cDTI acquisition is performed at the best cardiac phase, thereby avoiding long manual adjustments and scan iteration. Similar approaches to the TD scout have been used by Stoeck et al. [15] and Rapacchi et al. [25] to retrospectively identify acceptable trigger times for cardiac diffusion imaging.”

Figure R1: Examples of a TD scout acquisition and corresponding signal as a function of TD normalized over the RR interval. Representative cases in which it was possible (A) and not possible (B) to acquire cDTI during diastasis. The TD scout signal is reported as median (solid line) and [Q1 Q3] range (dashed line) measured across all volunteers as well as individual values (points) corresponding to each volunteer. The signal is computed across all images per volunteer after segmentation (see supplementary S1 Fig.) and after normalization by the cardiac phase with the mean highest signal. The colored boxes represent the temporal windows in which the cDTI has been acquired. Thus, the width of each box corresponds to the time from triggering to TE normalized with respect to the RR interval. Panel (C) shows the overall signal measured across all volunteers as a function of TD normalized over the RR interval. Boxplots show the distribution of TDs for early systole, late systole, and diastasis across all volunteers.

**R2.3) Similarly line 410 states that diastasis was the most common time available for imaging during diastasis. However, the methods section suggests that the trigger search window for diastole was limited to diastasis (line 170), making this statement in the discussion redundant.**

We thank the reviewer for noticing this. The sentence was edited as follow:

Discussion, P25-L468-475: “The cardiac phase imaged in diastole corresponded to diastasis, where LV motion pauses during filing before atrial systole begins. However, diastasis usually shortens as the heart rate increases and the beat-to-beat variation in cardiac motion may not respect the linear velocity/acceleration assumption behind the M1M2 waveform.“

**R2.4) Line 353, FA was slightly higher in early systole, but not significantly. In the prior sentence there is a similar magnitude in change in the eigenvalue ratio, which you say is not a change. I would probably avoid suggesting that there is any change in FA for consistency.**

We thank the reviewer for pointing this out. Yes, indeed a slightly higher FA was observed in early systole, but it was not found to be significant with respect to late systole or diastasis. We rephrased this sentence for the consistency of the section.

Results, P25-L468-475: “FA was also similar across cardiac phases, 0.36 [0.33, 0.40] A.U. at early systole, 0.33 [0.33, 0.35] A.U. at late systole and 0.35 [0.34, 0.36] A.U. at diastasis. “

**R2.5) Line 445: Results in ex-vivo fetal hearts are irrelevant when discussing HA mobility as the ex-vivo data is static.**

The reviewer is correct, this reference has been removed from the discussion.

**R2.6) Figure S1. There is a substantial artefact in these example images, which should be acknowledged and the source of which should be included in the legend. Was this artefact also present in the corresponding cDTI images?**

We thank the reviewer for this comment. No, the artifact was not present in the final images as shown in Figure 4 and is not usually present in the TD scout, which can be seen in the examples given in Figure 3. SNR is lower and some other non-motion related artifact may be present. The purpose of Figure S1 was only to graphically demonstrate how we calculated the TD scout signal curves. We agree with the reviewer, the attention of the reader focused quickly on the artifact and not on the purpose of the figure. We have updated Figure S1 using TD scout images with fewer artifacts while also mentioning in the Results section the possible presence of artifacts in the TD scout.

Results, P25-L468-475: “First, the subject specific TDs were determined by using the TD scout sequence. Because they were acquired during free-breathing (without a navigator) with less recovery time (TR~two heart beats instead of four), the TD scout images had lower signal and more non-motion related artifacts than the subsequent cDTI images. However, it was possible to visually identify motion related artifacts from the TD scout images. An example of the TD scout acquisition and the corresponding average signal per TD are displayed in Fig 3.”

Figure R2: Automatic estimation of the signal per cardiac phase imaged during the TD scout. A) Three images corresponding to three diffusion encoding directions x, y, and z are acquired during free breathing for each cardiac phase. B) The three diffusion weighted images are averaged together to generate a trace image. C) The trace image is then circularly cropped with a 48mm radius to only include the central zone of the image corresponding to the heart. D) The mean signal and standard deviation (SD) are computed using the cropped trace image for each cardiac phase.

**R2.7) Supplementary figure S4. I appreciate that the vertical scale of the E2A Bland-Altmann plots is chosen to be similar to that of the HAR, but the range of potential E2A values is much smaller and I would rather be able to see the distribution of E2A differences than maintain the consistent scale between angular measures. Please consider re-plotting with a different vertical scale.**

We agree with reviewer. The dynamic range of the E2A Bland-Altmann was too large. We have edited Figure S4 to use a vertical range of ±40° for HAR and ±10° for E2A.

Figure R3: Intra- and inter-observer variability in Helix Angle Range (HAR) (top) and E2 angle (E2A) (bottom) computed across two segmentations (intra-observer) and two observers (inter-observer). The intraclass correlation coefficient (ICC) for HAR was 76% overall and 84% for the inter-observer comparison. The intra-observer ICC was 72% for Observer-1 and 92% for Observer-2. The ICC for E2A was 99% overall and for the inter- and intra-observer analysis.

**R2.8-R2.20) Minor comments and typos. This list may not be complete.**

Line 59, additional full stop.

Line 76, redundant "imaging" after cDTI.

Line 188, inconsistent capitalisation of Eigenvectors (see next line). Also present elsewhere for other terminology.

Line 315, additional comma after 100.3] ˚

Throughout results, "paired-wised" should read "pair-wise"

Line 320, "between early [and] late"

Line 334: difference[s]

Line 446: Gotschy spelling mistake.

Line 468: metric[s]

Line 475: "across intra and inter trials" doesn't read well.

Line 498: intra quartile range -> interquartile range

Line 510: Consider removing "However"

Line 520: Define the positive direction used for DENSE displacements.

Thank you for noticing and pointing out these typos. We apologize for the last name spelling mistake. We did additional proofreading of the entire manuscript.
